# Notes and Comments

**Published:** 1895-10

**Authors:** 


### Notes and Comments.[1]

Self-Denial.—The key to success in any department of life is self denial. Idleness, laziness, wastefulness, come from lack of it; while industry, promptitude, economy, thrift, and a successful career are the result of it.—Neal Dow.

---

Bad Luck.—I never knew an early-rising, hard-working, prudent man, careful of his earnings, and strictly honest, who complained of bad luck. A good character, good habits, and iron industry are impregnable to the assaults of all the ill-luck that fools ever dreamed of.—Addison.

---

Devitalization of Highly Inflamed Pulp.—Professor Truman says, I have most satisfactory results from the use of iodoform in small quantities in connection with arsenic. So far as tried, there has not been a particle of pain in acute pulpitis.

---

Mosquito Bites.—A correspondent writes to the New York *Tribune* that an effectual and speedy cure for mosquito bites is aristol. The tip of the finger is moistened with water and a little of the powder taken and rubbed on the inflamed spot.

---

To mend Broken Plaster Casts.—Paint the broken surfaces over two or three times with very thick shellac varnish, and at each application burn out the alcohol over a flame. When the shellac is sufficiently soft, press the parts together, and hold in position till cool. It will be as strong as it was before broken.

---

After-Pains from Extraction of Exostosed Teeth.—When the bone has been distended and strained actual osteitis may result,

---

[1] The assistant editor solicits contributions for this department,—new methods, new remedies and formulas, or any short practical note which may prove of value to the practitioner or student. Address 1718 Walnut Street, Philadelphia.

with severe pain and inflammation. Dr. J. D. Thomas, the well-known specialist, says that the application of hot water will act like magic, relieving the congestion and diffusing the induration, establishing normal circulation through the parts.

---

Root-Canal Filling.—For doubtful root-canals Dr. Ottolengui prepares a gutta-percha cone by dipping waxed floss silk in chloro-percha and laying aside for chloroform to evaporate. Then fills canal with chloro-percha and carries silk gutta-percha cone to place, leaving projecting end in cavity. This is easily removed if trouble ensues.

---

Restoration of Hardened Rubber.—It is said that rubber goods which have become hardened by age may be restored to almost original softness by simply soaking in a water of ammonia diluted with twice its bulk of fresh water; and that this does not injure the rubber in any way, and restores the elasticity. Usually soaking for ten minutes to half an hour is quite sufficient. After drying, the whiteness may be restored by dusting well with chalk or kaolin.—*Dental Office and Laboratory.*

---

Method of mounting Disks and Points for Dental Engine.—Dr. T. F. Chupein claims that the best way to mount disks and points for the dental engine is with phosphate of zinc cement. Mix this to a creamy consistence. Drop a small quantity in the hole of the disk or point, and rub a little on the end of the mandrel. Place the mandrel point in the hole of the disk, and make it true by putting the shank of the mandrel through the disk setter. Let this remain till the cement gets perfectly hard, and you will find that the disk or point will never separate, which cannot be said of mounting these with gum shellac, as is generally done.

---

Rubber Dam for Pulp-Capping.—To the various well-known capping or non-conductive methods of procedure in pulp protection Dr. W. Storer How advises the interposition of a disk or rubber dam. The material is always at hand, and by folding a piece in sextant shape and with the scissors snipping off the point, an impromptu disk may be made to suit the cases. For example, cut two such disks. Having the cavity formed and dried for filling, the cavity floor and walls are to be lightly touched with a very little ball of cotton carrying about a drop of pure mastic varnish or other cavity lining. Then touching the cotton ball with the end of a curved canal broach, pick up the disk and delicately cover the cavity floor, to which the disk will stick; mix some cement, suitably soft; put a little on the centre of the second disk, lift it with a pair of fine-pointed foil tweezers and gently place its cement side against the other disk, and with a ball burnisher softly spread the cement under the second disk to completely cover the cavity floor and partly cover the cavity walls. Allowing some minutes for the setting of the cement, the remaining area of the cavity can be filled with whatever material may be preferred.

